# Disease Threat and the Functional Flexibility of Ingroup Derogation

**DOI:** 10.3389/fpsyg.2019.02030

**Published:** 2019-08-30

**Authors:** Qi Wu, Shuang Yang, Ping Zhou

**Affiliations:** Cognition and Human Behavior Key Laboratory of Hunan Province, Department of Psychology, Hunan Normal University, Changsha, China

**Keywords:** ingroup derogation, behavioral immune system, disease threat, smoke detection principle, functional flexibility principle

## Abstract

While the findings from previous studies directly relate the ingroup derogation phenomenon to the evolved response of the behavioral immune system, there are three major limitations in the previous studies on the functional flexibility of ingroup derogation. The present study further investigated the functional flexibility of ingroup derogation by conducting three behavioral experiments on Chinese participants. In Experiment 1, we tested whether exposing to situational disease primes leads to an exaggerated ingroup derogation attitude by adopting a more rigorous control. In Experiment 2, we manipulated the source of disease threats to test whether the ingroup derogation mechanism adjusts its response according to the specific perceived vulnerabilities to the disease threats posed by ingroup and outgroup members. In Experiment 3, we tested whether recent illness promotes the expression of ingroup derogation attitudes. Results of the three experiments consistently showed that, the Chinese participants adjusted their ingroup derogation attitudes according to the external environmental disease cues (Experiments 1 and 2) and the internal physiological disease cues (Experiment 3). The results also showed that the ingroup derogation mechanism was sensitive to the specific perceived vulnerabilities to the ingroup disease threat and the outgroup disease threat (Experiment 2). Taken together, these results support the evolutionary hypothesis of ingroup derogation and suggest that the ingroup derogation found in East Asian cultures could be accounted by a functionally flexible disease-avoidance mechanism.

## Introduction

In the long history of human species, group living is essential to one’s reproductive fitness. To simplify the social world’s complex structure, people regularly parse the social world into “us” and “them” (Hewstone et al., 2002). They usually display a systematic tendency to favor one’s own membership group (the ingroup) over a non-membership group (the outgroup) (Hewstone et al., 2002). This bias is referred as ingroup favoritism (or ingroup bias) in social psychology. It can be found among the actual social groups in which there are real differentiations between “us” and “them” (Sosis and Ruffle, 2003; Whitt and Wilson, 2007; Rand et al., 2009; Petersen, 2017). It also can be found among the artificial minimal social groups (i.e., by using minimal group paradigm) in which only a heuristic cue of the differentiation between “us” and “them” is provided (Tajfel et al., 1971; Brewer, 1979; Bernstein et al., 2007; Paladino and Castelli, 2008; Montalan et al., 2012; Makhanova et al., 2015).

Although the mainstream psychology has documented the universal tendency of ingroup favoritism, a similar but completely opposite phenomenon of ingroup derogation (or sometimes be referred as outgroup favoritism) has also been reported. That is, some participants were found to show a preference for outgroup members relative to ones’ ingroup members (Jost et al., 2002; Ma-Kellams et al., 2011; Zhao et al., 2012; Liu et al., 2015; March and Graham, 2015; Wu et al., 2015, 2016; Barker and Barclay, 2016; Zuo et al., 2018; Bettache et al., 2019). This counterintuitive bias was initially found in minorities or inferior social groups (Allport, 1958; Jost et al., 2002; Livingston, 2002; Rudman et al., 2002; Ashburn-Nardo et al., 2003; Umphress et al., 2008; March and Graham, 2015; Axt et al., 2018). Studies also revealed that participants rated the deviant ingroup members more negatively compared with their outgroup counterparts (i.e., the black sheep effect; see Marques et al., 1988; Reese et al., 2013; Kunstman et al., 2016; Bettache et al., 2019). In addition, studies also showed that, in East Asian cultures, even though the participants were not minorities or deviants, they still seemed to possess a general, status irrelevant, and pervasive negative posture toward ingroup members (Jahoda et al., 1972; Hewstone and Ward, 1985; Lee and Ottati, 1993, 1995; Diener et al., 1995; Heine and Lehman, 1997; Endo et al., 2000; Snibbe et al., 2003; Cuddy et al., 2009; Ma-Kellams et al., 2011; Zhao et al., 2012; Liu et al., 2015; Wu et al., 2015, 2016; Zuo et al., 2018; Xie et al., 2019). For example, researchers found that the Chinese implicitly associated Westerners with more positive traits and more civilized behaviors than their own ethnic group members (Ma-Kellams et al., 2011; Liu et al., 2015), and they were more prone to make outgroup-favoring and ingroup-disfavoring attributions (Hewstone and Ward, 1985). It was also reported that the Chinese perceived the faces and names of outgroup members as more beautiful and better (Zhao et al., 2012; Wu et al., 2016), and were more inclined to cooperate with outgroup members (Wu et al., 2015, 2016), when actually both the ingroup and outgroup members were having the same neutral average looks.

Few studies have examined the causal origins of ingroup derogation. Researchers found that it was difficult to explain ingroup derogation in terms of proximate cause (Ma-Kellams et al., 2011; Zhao et al., 2012; Wu et al., 2015, 2016). In addition, the existence of ingroup derogation is also a paradox in an evolutionary sense (Wu et al., 2015, 2016). Individuals who preferred ingroups should have been favored by natural selection, whereas individuals displaying ingroup disfavoring tendencies should be eliminated from the gene pool over time (Brewer, 2007; Fincher and Thornhill, 2008a,b, 2012a,b; Van Vugt and Park, 2009; Schaller and Murray, 2010; Neuberg et al., 2011; Schaller and Neuberg, 2012; Thornhill and Fincher, 2014; Neuberg and Schaller, 2016; Ji et al., 2019). Thus, from an evolutionary perspective, preference for outgroup members should be considered as a maladaptation, which makes it difficult of explain the prevalence and persistence of ingroup derogation.

### Behavioral Immune System and Ingroup Derogation

The behavioral immune system is composed of mechanisms that evolved as a means of inhibiting contact with disease-causing parasites and facilitating behaviors that minimized infection risk and enhanced fitness (Schaller and Neuberg, 2012; Schaller et al., 2015; Murray and Schaller, 2016). Recent studies have demonstrated that this system has unique consequences for many aspects of human social cognition and behaviors (Fincher and Thornhill, 2008a,b, 2012a,b; Van Vugt and Park, 2009; Schaller and Murray, 2010; Neuberg et al., 2011; Schaller and Neuberg, 2012; Thornhill and Fincher, 2014; Schaller et al., 2015; Murray and Schaller, 2016; Neuberg and Schaller, 2016; Bonin et al., 2019; Mullett et al., 2019). One of the main impacts of the behavioral immune system is its involvement in the emergence of the ubiquitous tendency of ingroup favoritism. Researchers proposed that, since the physiological immune system of an organism is primarily shaped by the local pathogen ecology, the outgroup members may often harbor the novel pathogens that are infectious to an individual and its immunologically similar ingroup members (Fincher and Thornhill, 2008a,b, 2012a,b; Schaller and Murray, 2010; Thornhill and Fincher, 2014). Therefore, under ecological conditions of high disease stress, a psychological mechanism facilitating the aggregation of ingroup members but inhibiting contacts with outgroup members is adaptive for its functional value of avoiding novel pathogens and minimizing local infectious risks and thus should be favored by natural selection^1^ (Fincher and Thornhill, 2008a,b, 2012a,b; Van Vugt and Park, 2009; Schaller and Murray, 2010; Neuberg et al., 2011; Schaller and Neuberg, 2012; Thornhill and Fincher, 2014; Schaller et al., 2015; Murray and Schaller, 2016; Neuberg and Schaller, 2016; Ji et al., 2019; Zakrzewska et al., 2019).

Not only the ingroup favoritism can be explained by the functionality of behavioral immune system, but also the existence of ingroup derogation is possible to be accounted by the system’s pathogen defense function. Recently, researchers proposed a novel evolutionary hypothesis to explain the general ingroup derogation tendency found in East Asian cultures (Wu et al., 2015). Specifically, it was proposed that the assumption of the disease threat posed by outgroup members was much greater than the disease threat posed by ingroup members is problematic. For example, if there are outbreaks of some emerging diseases in the local habitat of ingroup members, or somehow the pathogen load within the local habitat of ingroup members become much higher than the pathogen load within the local habitat of outgroup members (such as environmental change), it would be much more easier to catch an infectious disease *via* an ingroup member than *via* an outgroup member. Under such circumstances, it would be more adaptive to derogate, to dislike, and to avoid ingroup members than to bond with them. If such situations did occur recurrently in the evolutionary history of the human race, a psychological mechanism that facilitates ingroup derogation responses under particular ecological conditions should be favored by nature selection. Thus, this hypothesis suggests that the East Asians derogate their ingroups because they are responding to heuristic cues indicating the disease threat incurred by the ingroup members has become stronger than the disease threat incurred by the outgroup members (Wu et al., 2015).

Some indirect evidence suggests that this hypothesis could be supported. For example, besides being an area where ingroup derogation attitudes are prevalent (Ma-Kellams et al., 2011; Zhao et al., 2012; Liu et al., 2015; Wu et al., 2015, 2016; Zuo et al., 2018), evidence also indicates that China may have higher pathogen prevalence than other areas (e.g., Europe) by both historical and contemporary measures (Fincher et al., 2008; Chang et al., 2011). Theoretical works also suggest that in face of high pathogen load, ingroup investment is not optimal and thus should be reduced (Thornhill and Fincher, 2014), and they also suggest that individuals may prefer to cooperate with outgroup members instead of ingroup members if the infection risk associated with outgroup members is low (Brown et al., 2016) or when the infection risk associated with ingroup members is high (Hu et al., 2018). Consistent with these theoretical works, empirical studies also reported that the associations between pathogen load and ingroup favoritism attitudes were found to be inconsistent or to be none. Studies also revealed that the relationship between these two variables is better to be described by a quadratic function than by a simple linear model (i.e., the ingroup favoritism drops when the pathogen load rises to a certain level; e.g., Fincher and Thornhill, 2012a,b; Cashdan and Steele, 2013; Hruschka and Henrich, 2013; Talhelm et al., 2014). Studies on generalized social trust also revealed that both the ingroup trust and outgroup trust are negatively associated with local pathogen load (Aarøe et al., 2016), and the outgroup trust actually rises when the local pathogen load exceeds a certain threshold (Zhang, 2018).

Direct evidence for this hypothesis also has been obtained. Researchers found that mere social categorization alone is already sufficient to elicit ingroup derogation among Chinese participants, which suggests that ingroup derogation follows the smoke detector principle of behavioral immune system (i.e., the behavioral immune system responds to heuristic cues which imply the presence of diseases and thus is prone to make false-positive errors; Wu et al., 2015, 2016). In addition, they also found that the ingroup derogation attitude was positively associated with the perceived vulnerability to diseases, and such an intergroup bias was found to be exaggerated if there were diseases cues in the immediate environment (Wu et al., 2015). Further evidence indicates that the positive correlation between the perceived vulnerability to diseases and ingroup derogation among mainland Chinese was mainly driven by the negative correlation between ingroup attitude and perceived vulnerability to diseases, and the results also showed that the Chinese participants responded more strongly to the diseases cues mediated by ingroup members rather than to the diseases cues mediated by outgroup members (Wu et al., 2015). These results suggest that the ingroup derogation follows the functional flexibility principle of behavioral immune system (i.e., under circumstances in which individuals are easy to be infected or merely perceive themselves to be vulnerable to infection, the activation of behavioral immune system is stronger; Van Vugt and Park, 2009; Schaller et al., 2015; Murray and Schaller, 2016). Taken together, these results suggest that the ingroup derogation in East Asian cultures is related to a specialized response of behavioral immune system and it is designed to deal with a special ecological condition in which the greater threat of diseases is incurred by ingroup members (instead of by outgroup members).

### The Current Study

Although the current evidence seems to be consistent with the evolutionary hypothesis of ingroup derogation, it is still necessary to be cautious and consider the evidence as preliminary. Specifically, there are three major limitations in the current evidence concerning the functional flexibility of ingroup derogation (Wu et al., 2015). First, the functional flexibility principle dictates that the behavioral immune system should be sensitive to individuals’ apparent vulnerability to pathogen threat and modulates the threat-minimizing responses accordingly (Van Vugt and Park, 2009; Schaller et al., 2015; Murray and Schaller, 2016). Therefore, as an evolved response, the ingroup derogation attitude should be adjusted according to external disease cues (Wu et al., 2015). However, until now, researchers had only examined the effects of pathogen threat on ingroup derogation attitudes by employing a no-threat control (Wu et al., 2015). This was not rigorous enough to completely rule out other plausible explanations. For example, it is possible that the observed effects of pathogen threat were actually caused by the byproduct of unspecific emotional arousal. Second, in the study of Wu et al. (2015), researchers had only investigated the functional flexibility of ingroup derogation under situations in which the relative risk of infection between ingroup and outgroup was fixated. However, if ingroup derogation is indeed an evolved response of the behavioral immune system, the ingroup derogation mechanism should be able to adjust its responses according to the specific perceived vulnerabilities to the ingroup disease threat and the outgroup disease threat. Third, the activation of the behavioral immune system has been shown to be closely related to the biological immune system (Murray et al., 2019). If ingroup derogation is indeed a functionally flexible response of the behavioral immune system, the ingroup derogation attitude should not only be adjusted when there were external disease cues. It also should be more exaggerated when the biological immune system is inhibited since such a condition indicates a heightened susceptibility to diseases (Miller and Maner, 2011; Lund and Miller, 2014; Kandrik et al., 2017; Oaten et al., 2017; Gassen et al., 2018; Bradshaw et al., 2019; Murray et al., 2019). However, this important feature of ingroup derogation tendency has not been investigated in the previous study (Wu et al., 2015) in which the researchers had only examined the roles of subjective and situational disease cues in the expression of ingroup derogation.

To address these limitations, we conducted three experiments in the current study to further investigate the functional flexibility of ingroup derogation. We mainly focused on the ingroup derogation among mainland Chinese. In Experiment 1, we tested whether exposing to a situational disease prime leads to an exaggerated ingroup derogation attitude by adopting a more rigorous control. In Experiment 2, we manipulated the source of disease threat to test whether the ingroup derogation mechanism can adjust its response according to the specific perceived vulnerabilities to the different disease threats posed by ingroup and outgroup members. Since being recently ill temporarily lowers the physiological immune function and consequently activates the behavioral immune system (Miller and Maner, 2011; Lund and Miller, 2014; Kandrik et al., 2017; Oaten et al., 2017; Murray et al., 2019), we also tested whether recent illness promotes the expression of ingroup derogation attitudes in Experiment 3.

According to the evolutionary hypothesis of ingroup derogation, mere social categorization alone – a heuristic cue that implies the differentiation between “us” and “them” – should be sufficient to bring the bias of ingroup derogation (i.e., smoke detector principle; e.g., Wu et al., 2015, 2016). As a wide-accepted paradigm to study intergroup bias in the laboratory, the minimal group paradigm categorizes people into arbitrary social categories or groups, such as whether they have a “red” personality type or a “green” personality type based on bogus personality tests, which provides group-categorization heuristics to one’s actual social group membership (Tajfel et al., 1971; Brewer, 1979; Bernstein et al., 2007; Paladino and Castelli, 2008; Makhanova et al., 2015; Wu et al., 2015, 2016). Studies employing this paradigm have shown strong cognitive, motivational, and behavioral differences in responses to these arbitrarily constructed ingroups and outgroups,^2^ which were very similar to the responses elicited by actual social groups (e.g., Tajfel et al., 1971; Brewer, 1979; Bernstein et al., 2007; Paladino and Castelli, 2008; Makhanova et al., 2015; Wu et al., 2015, 2016; Zuo et al., 2018; Dang et al., 2019). Therefore, following the studies of Wu et al. (2015) and Wu et al. (2016), we also employed the minimal group paradigm to elicit the ingroup derogation phenomenon.

Studies on ingroup favoritism have shown that participants incline to affiliate to and cooperate with their ingroup members (Sosis and Ruffle, 2003; Ruffle and Sosis, 2006; Whitt and Wilson, 2007; Rand et al., 2009; Van Vugt and Park, 2009; Yamagishi and Mifune, 2009; Neuberg et al., 2011; Schaller and Neuberg, 2012; Fincher and Thornhill, 2012a,b), while studies of ingroup derogation found the reversed patterns. For example, it was reported that the mainland Chinese were more inclined to cooperate with outgroup members if they were asked to choose their partners based on the facial information and group membership, while actually both the ingroup and outgroup members were having the same neutral average looks (Wu et al., 2015, 2016). Following these studies, we used the degree of acceptance (i.e., acceptance of a specific group member as a partner to work with) as the measure of participants’ preference for a specific group membership in the present study. If the participants were more inclined to work with outgroup members, then they harbored an ingroup derogation attitude. If the pattern was reversed, then they displayed an ingroup favoritism attitude (for same measures of intergroup bias, see Navarrete and Fessler, 2006; Wu et al., 2015, 2016).

## Experiment 1

As a specialized response of behavioral immune system, the ingroup derogation mechanism should follow the functional flexibility principle. This means that the activation of ingroup derogation mechanism should be promoted when there are cues of diseases in the immediate environment. The previous study (Wu et al., 2015) has shown that Chinese participants displayed more exaggerated ingroup derogation attitudes when they were placed in a disease environment (i.e., finishing the experiment with a very dirty keyboard) or when the ingroup and outgroup members were both displaying the cues of diseases. However, researchers had only compared the effects of pathogen threat to a no-threat control in the previous study (Wu et al., 2015). This kind of control is not rigorous enough to completely rule out other alternative explanations. For example, the effects of pathogen threat found by Wu et al. (2015) might actually be caused by the high arousal state created by pathogen threat rather than by the specific disease features of pathogen threat. It is also possible that the ingroup derogation mechanism is nonspecifically responding to all kinds of threats rather than specifically responding to the disease threat. To rule out these possibilities, we extended and replicated the study of Wu et al. (2015) by adopting a more rigorous control in Experiment 1. Specifically, following prior research (Park et al., 2007; Miller and Maner, 2012; Wu and Chang, 2012; Lund and Miller, 2014; Makhanova et al., 2015; Nussinson et al., 2018; Wang and Ackerman, 2019), we experimentally primed Chinese participants with either disease-connoting images or images of non-disease-related threats. Consistent with previous studies on ingroup derogation (Zhao et al., 2012; Wu et al., 2015, 2016), we measured the ingroup derogation attitudes of Chinese participants by asking them to finish a face appraisal task in which only the facial information and group membership were provided. Given that Chinese participants were responding to a special ecological condition in which the greater threat of disease was posed by ingroup members, immediate disease cues in the environment should elicit more avoidance responses to ingroup members than to outgroup members in these participants. Therefore, we predicted that, compared with the non-disease-threat priming, Chinese participants should exaggerate their ingroup derogation attitudes after the disease prime even when the overall affective valence and arousal were well matched between the two different priming conditions.

### Method

#### Participants and Design

G*Power Version 3.1.9.2 software (Faul et al., 2009) was used to acquire an *a priori* estimate of the required sample size. Using the parameters (power = 0.99, effect size *f* = 0.21,^3^ *α* = 0.05; Richard et al., 2003) and giving the current experimental design, the analysis estimated a sample size of 108. We finally recruited a total of 120 Chinese undergraduate or postgraduate students (60 males and 60 females, aged 18–25 years). Sensitivity power analysis indicated that, the minimal detectable effect (power = 0.99) for this sample size is *f* = 0.197. This experiment was carried out in accordance with the recommendations of the IRB of the Institute of Psychology, Hunan Normal University, with written informed consent from all participants. All participants gave written informed consent in accordance with the Declaration of Helsinki. The protocol was approved by the IRB of the Institute of Psychology, Hunan Normal University.

A 2 (category label: ingroup, outgroup) × 2 (priming condition: disease prime, control) mixed-model experimental design was used, with priming condition being the between-subjects factor and category label being the within-subjects factor.

#### Materials and Procedure

Following previous studies (Bernstein et al., 2007; Paladino and Castelli, 2008; Makhanova et al., 2015; Wu et al., 2015, 2016), a bogus personality test was employed to create the minimal groups. This test consisted of 40 questions taken from the Eyesenck Personality Questionnaire (Eysenck and Eyesenck, 1975). The computer ostensibly analyzed participants’ responses and then randomly informed the participants that they had either a “red” or “green” personality type. Participants were then told that each personality type was not necessarily better than the other personality type and the purpose of this experiment was to investigate psychological differences between these two different personality types. Given no further explanation, participants were given a green or red identity tag to wear, and told it was to identify their particular personality type (see Wu et al., 2015, 2016, for the same procedure).

Eighty gray-scale facial images of Chinese adults displaying neutral facial expressions were chosen as the stimuli (directly adopted from Zhao et al., 2012). These images were completely novel to all participants and they consisted of two image sets (with 40 faces in each set) which were matched on the degrees of beauty (all were average looking faces; Zhao et al., 2012) and acceptance (Wu et al., 2015). The facial stimuli were presented in the same way as in Wu et al. (2015) and Wu et al. (2016). Each face was presented in the center of the screen and a label of personality type (red or green) was placed at the top of the background in order to label the face. The background color of the screen was set to be identical to the personality label (red or green). These two image sets were counterbalanced across background color (and its personality label) on a between-subjects basis. Thus, each image set has an equal probability of being labeled as ingroup or outgroup members.

Participants were randomly assigned either to a disease priming condition or to a control condition. Participants in each condition were instructed that they would take a computerized personality test at first. Then, they were told that they were going to complete another unrelated task before completing the “formal experiment.” Following prior researches (Park et al., 2007; Miller and Maner, 2012; Wu and Chang, 2012; Lund and Miller, 2014; Makhanova et al., 2015; Nussinson et al., 2018; Wang and Ackerman, 2019), participants in the disease priming condition watched a slideshow consisting of 10 images that portrayed information about germs, infections, and other diseases. Participants in the control condition watched a slideshow of 10 images portraying information about common accidents and hazards (e.g., car accidents, air crash) that were non-disease related threats. Each image was shown for 6 s and participants were asked to watch closely to answer questions about them. Each participant was asked to use 9-point scales to rate the valence (1 = “very unpleasant” and 9 = “very pleasant”) and arousal (1 = “very calming” and 9 = “very arousing”) of his/her current emotional state after slide watching. Then, participants were told that they had to complete the “formal experiment” which was a face appraisal task. Participants were instructed that they would view faces on the screen, and that the background color and the label displayed on the top of the screen would denote the target’s personality type. Their task was to rate “to what extent would you want to work together with the person shown on the screen in the next experiment” on an 8-point scale (1 = “definitely not” to 8 = “definitely like to”) for these faces. The faces were presented one at a time, and each face remained on the screen until the response was made. Faces were randomly presented for each participant.

### Results and Discussion

Independent *t*-tests showed that there were no significant differences between the two priming conditions for the overall affective valence [disease prime: *M* = 1.85, SD = 1; control: *M* = 2.12, SD = 1.12; *t*(118) = −1.37, *p* = 0.17] and arousal [disease prime: *M* = 6.57, SD = 1.59; control: *M* = 6.2, SD = 1.15; *t*(107.39) = 1.45, *p* = 0.15]. Thus, the manipulation was successful at creating intended differences in threat contents but without creating differences in overall affect.

**Figure 1:**
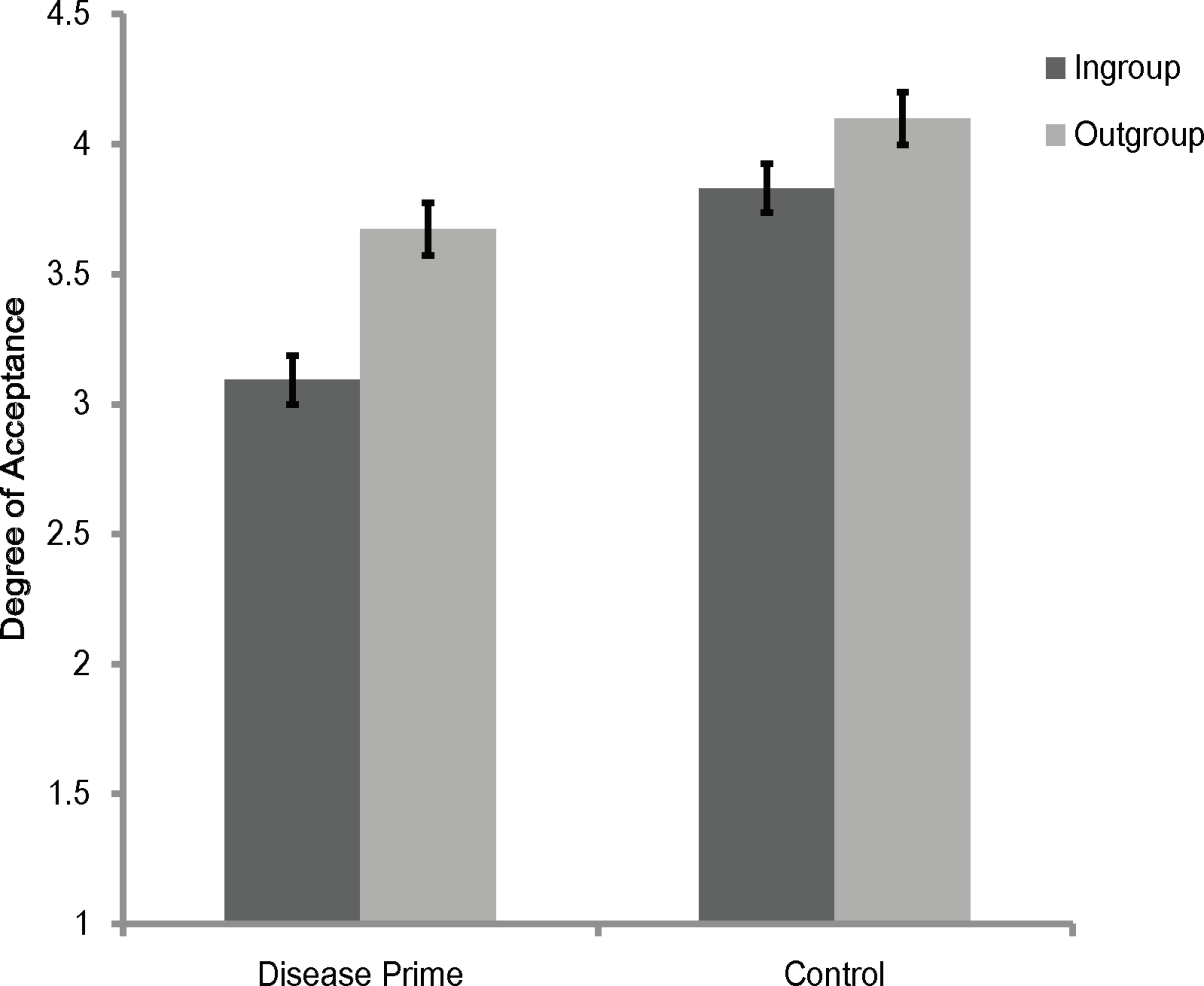
Degree of acceptance of faces labeled as ingroup members and outgroup members in Experiment 1. Error bars represent standard errors.

A 2 (category label) × 2 (priming condition) mixed model analysis of variance (ANOVA) on rating scores of face appraisal task showed that the main effect of category label was significant [*F*(1, 118) = 61.95, *p* < 0.001, $\eta_{p}^{2}$ = 0.344], and the main effect of priming condition [*F*(1, 118) = 20.88, *p* < 0.001, $\eta_{p}^{2}$ = 0.15] and the interaction between category label and priming condition [*F*(1, 118) = 8.37, *p* = 0.005, $\eta_{p}^{2}$ = 0.07] were significant. Simple effects analysis showed that participants under all priming conditions were consistently more inclined to affiliate with outgroup members than with ingroup members [disease prime: *F*(1, 118) = 57.93, *p* < 0.001, $\eta_{p}^{2}$ = 0.33; control: *F*(1, 118) = 12.39, *p* = 0.001, $\eta_{p}^{2}$ = 0.1] (see Figure 1). It also revealed that participants in the disease prime condition showed less favorable attitudes toward both ingroup [*F*(1, 118) = 30.61, *p* < 0.001, $\eta_{p}^{2}$ = 0.21] and outgroup [*F*(1, 118) = 8.84, *p* = 0.004, $\eta_{p}^{2}$ = 0.07] members than participants in the control condition (see Figure 1).

To further illustrate the interaction between category label and priming condition, rating scores of outgroup members in the face appraisal task were subtracted by that scores of ingroup members to create a composite score of ingroup derogation, and we subjected this score to a one-way ANOVA (with the priming condition being the independent variable). The results showed that the main effect of priming condition was significant [*F*(1, 118) = 8.37, *p* = 0.005, $\eta_{p}^{2}$ = 0.07], with participants showing more ingroup derogation attitudes in the disease prime condition (*M* = 0.58, SD = 0.63) than in the control condition (*M* = 0.27, SD = 0.55).

In sum, by adopting more a rigorous control, Experiment 1 replicated the results of previous studies of ingroup derogation (e.g., Zhao et al., 2012; Wu et al., 2015, 2016). These results of Experiment 1 indicated that, compared with a non-disease-threat prime, participants displayed stronger ingroup derogation attitude after a disease prime even with the overall affective valence or arousal was well controlled, and this effect was caused by the more exaggerated ingroup avoiding responses. They suggest that ingroup derogation mechanism is a mechanism that responds exclusively to the threat of disease and thus support the hypothesis that ingroup derogation found in East Asian cultures is an evolved response of the behavioral immune system.

## Experiment 2

The ingroup derogation mechanism should not be a simple and rigid mechanism that can only be more activated when there are cues of diseases in the immediate environment (as shown in Experiment 1). As a flexible mechanism, it has to be sensitive to the specific perceived vulnerabilities to ingroup/outgroup disease threats to better adjust to the changing benefits and costs associated with approaching/avoiding the ingroup or outgroup members. Therefore, to facilitate the ingroup avoiding response, the ingroup derogation attitude should become more exaggerated when there are cues of diseases indicating that the ingroup members are infectious, whereas a reversed pattern should be observed when the environmental cues indicate that the outgroup members are very infectious. These possibilities were tested in Experiment 2. Specifically, we predicted that the Chinese participants would exaggerate their ingroup derogation attitudes after watching a disease prime in which the ingroup members are depicted as infectious (compared with a disease-related control prime), whereas participants receiving a disease prime which depicts the outgroup members as infectious should reduce their ingroup derogation attitudes accordingly.

### Method

#### Participants and Design

G*Power Version 3.1.9.2 software (Faul et al., 2009) was used to acquire an *a priori* estimate of the required sample size. Using the parameters (power = 0.95, effect size *f* = 0.21, *α* = 0.05; Richard et al., 2003) and giving the current experimental design, the analysis estimated a sample size of 93. We finally recruited a total of 90 Chinese undergraduate students (46 males and 44 females, aged 18–22 years). Sensitivity power analysis indicated that, the minimal detectable effect (power = 0.95) for this sample size is *f* = 0.21. This experiment was carried out in accordance with the recommendations of the IRB of the Institute of Psychology, Hunan Normal University, with written informed consent from all participants. All participants gave written informed consent in accordance with the Declaration of Helsinki. The protocol was approved by the IRB of the Institute of Psychology, Hunan Normal University.

A 2 (category label: ingroup, outgroup) × 3 (disease prime condition: ingroup disease prime, outgroup disease prime, control) mixed-model experimental design was used, with disease prime condition being the between-subjects factor and category label being the within-subjects factor.

#### Materials and Procedure

Participants were randomly assigned to one of the three disease prime conditions. Then participants were instructed to finish a bogus personality test (as described in Experiment 1) in order to create the minimal groups. After that, participants were instructed that they were going to complete another unrelated task before completing the “formal experiment.” Specifically, participants were instructed that they would view several medical cases and they had to watch closely in order to answer several questions about these cases after finishing the “formal experiment.” Participants in the ingroup and outgroup disease prime conditions were further instructed that these medical cases were selected from the persons that were identical to (ingroup disease prime condition) or opposite to (outgroup disease prime condition) their own personality type. They were also told that the background color of the screen and the label displayed on the top of the screen would denote the personality type of the target person. Then, participants under all disease prime conditions directly watched the corresponding disease primes.

In the control condition, the disease-related control prime consisted of eight slides displaying images of Chinese adults (half of the targets were male, while the other half were female) who were infected with skin diseases (e.g., herpes, scabies, tinea corporis). The images within each slide consisted of one facial image (with a neutral facial expression) of the target person and one image of the infected part of the target’s body. The images were placed on the upper half of the screen, with the facial image being placed on the left and the image of the infected part being placed on the right. The background of the slide was set to be gray, and a paragraph of text was placed on the bottom of screen to describe the target’s symptoms. Twenty participants who did not participate in the formal experiment rated the contents of these disease primes. They had to rate that whether these slides portrayed relevant information about disease threat, sexual activity, and other threats (i.e., aggression, deception, and natural disaster) on 7-point scales (−3 = “definitely not,” 0 = “I’m not sure,” 3 = “definitely yes”). One sample *t*-test showed that the disease prime in control condition clearly conveyed information about diseases (*M* = 2.76, SD = 0.33), *t*(19) = 37, *p* < 0.001, but they did not contain relevant information about sexual activity (*M* = −2.73, SD = 0.5), *t*(19) = −24.25, *p* < 0.001, and other threats (*M* = −2.61, SD = 0.43), *t*(19) = −27, *p* < 0.001. The contents of disease primes under ingroup and outgroup disease prime conditions were identical to those in control condition, but with the background color being set to be identical (ingroup disease prime) or opposite (outgroup disease prime) to the color of the participant’s assigned personality type (red or green). A label (red personality type or green personality type) which was identical to the background color was placed on the top of the slide in order label the target person depicted in the slide. Each slide was presented for 20 s under all disease prime conditions.

After the disease threat priming, participants were asked to finish the “formal experiment” which was a face appraisal task (as described in Experiment 1). Facial images of the target persons in the disease primes were not included in the 80 facial stimuli (as described in Experiment 1) of the face appraisal task.

### Results and Discussion

**Figure 2:**
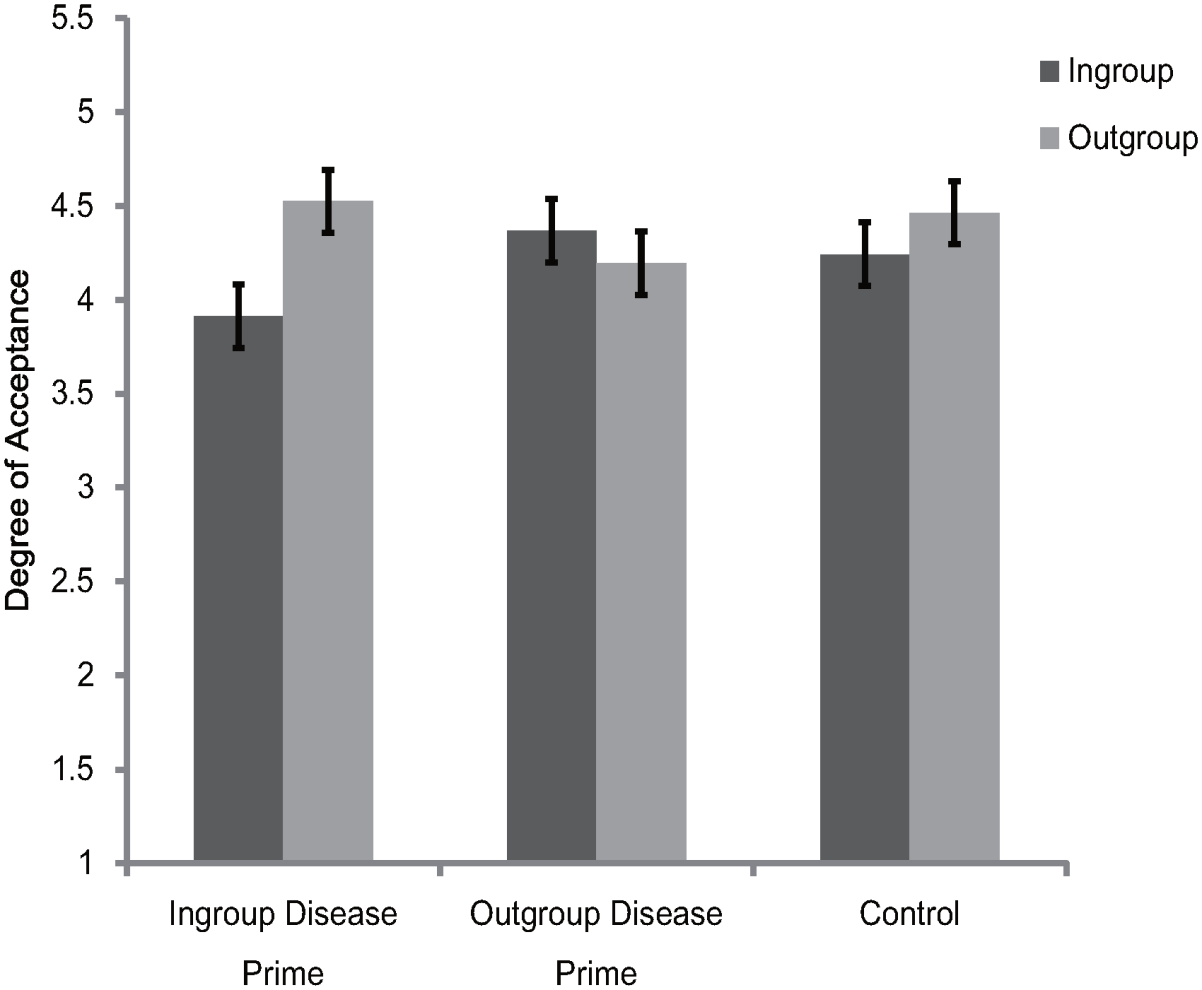
Degree of acceptance of faces labeled as ingroup members and outgroup members in Experiment 2. Error bars represent standard errors.

Rating scores for ingroup and outgroup members were subjected to a 2 (category label) × 3 (disease prime condition) mixed-model ANOVA. The results showed that the main effect of category label [*F*(1, 87) = 13.03, *p* = 0.001, $\eta_{p}^{2}$ = 0.13] and the interaction between category label and disease prime condition [*F*(1, 87) = 13.86, *p* < 0.001, $\eta_{p}^{2}$ = 0.24] were significant. The main effect of disease prime condition was not significant [*F*(2, 87) = 0.17, *p* = 0.84, $\eta_{p}^{2}$ = 0.004]. Further simple effects analysis showed that participants in the ingroup disease prime condition [*F*(1, 87) = 33.64, *p* < 0.001, $\eta_{p}^{2}$ = 0.28] and participants in the control condition [*F*(1, 87) = 4.4, *p* = 0.04, $\eta_{p}^{2}$ = 0.05] were more inclined to affiliate with outgroup members than with ingroup members, but there were no significant differences between the ingroup and outgroup attitudes [*F*(1, 87) = 2.7, *p* = 0.1, $\eta_{p}^{2}$ = 0.03] for participants under the outgroup disease prime condition (see Figure 2). The results also showed that the effects of disease prime condition were not significant for ingroup members [*F*(2, 87) = 1.93, *p* = 0.15, $\eta_{p}^{2}$ = 0.04] and outgroup members [*F*(2, 87) = 1.1, *p* = 0.34, $\eta_{p}^{2}$ = 0.03].

To further illustrate the interaction between category label and disease prime condition, an ingroup derogation score (as described in Experiment 1) was created. The one-way ANOVA showed that the differences of ingroup derogation scores among the three disease prime conditions were significant [*F*(1, 87) = 13.86, *p* < 0.001, $\eta_{p}^{2}$ = 0.24]. Further *post hoc* comparisons (Bonferroni) showed that, participants in the ingroup disease condition were more likely [*t*(87) = 2.6, *p* = 0.03] to derogate their ingroup member (*M* = 0.61, SD = 0.58) than participants in the control condition (*M* = 0.22, SD = 0.42). In addition, the results also showed that, compared with participants in the ingroup disease prime [*t*(87) = 2.65, *p* = 0.03] and control prime [*t*(87) = 5.26, *p* < 0.001] conditions, participants in the outgroup disease prime condition endorsed less ingroup derogation attitudes (*M* = −0.17, SD = 0.69).

Collectively, the results of Experiment 2 indicated that the Chinese participants exaggerated their ingroup derogation attitudes after receiving the ingroup disease prime, but the tendency of ingroup derogation was eliminated after receiving the outgroup disease prime. These results suggest that ingroup derogation is a functional flexible mechanism which can adjust its response according to the specific perceived vulnerabilities to disease threats posed by ingroup and outgroup members. These results were consistent with our prediction and thus provide support for the evolutionary hypothesis of ingroup derogation.

## Experiment 3

To protect the body from severe tissue damage and facilitate the recovering from recent infections, our biological immune system produces an anti-inflammatory response after being recently sick (Mocellin et al., 2003), which temporarily inhibits the physiological defenses against new pathogens (Jakab, 1985; LeVine et al., 2001; van der Sluijs et al., 2004) and consequently promotes the activation of behavioral immune system (Miller and Maner, 2011; Lund and Miller, 2014; Kandrik et al., 2017; Oaten et al., 2017; Murray et al., 2019). Therefore, as a functionally flexible response of the behavioral immune system, the ingroup derogation attitude should not only be exaggerated when there are external cues of diseases in the immediate environment (as shown in Experiments 1 and 2). It also should be more exaggerated when the responses of the biological immune system are inhibited since such a condition indicates a heightened susceptibility to diseases. In Experiment 3, we examined whether recent illness would lead Chinese participants to exaggerate their ingroup derogation attitudes.

### Method

#### Participants and Design

G*Power Version 3.1.9.2 software (Faul et al., 2009) was used to acquire an *a priori* estimate of the required sample size. Using the parameters (power = 0.99, effect size *f* = 0.21, *α* = 0.05; Richard et al., 2003) and giving the current experimental design, the analysis estimated a sample size of 108. A total of 122 Chinese undergraduate or postgraduate students (60 males and 62 females, aged 18–24 years) were finally recruited by advertisement. Specifically, 60 participants had been sick within the previous week (recently sick), and 62 participants had not been recently sick (i.e., the last time they had been sick was more than 1 week ago). Sensitivity power analysis indicated that, the minimal detectable effect (power = 0.99) for this sample size is *f* = 0.196. This experiment was carried out in accordance with the recommendations of the IRB of the Institute of Psychology, Hunan Normal University, with written informed consent from all participants. All participants gave written informed consent in accordance with the Declaration of Helsinki. The protocol was approved by the IRB of the Institute of Psychology, Hunan Normal University.

A 2 (category label: ingroup, outgroup) × 2 (illness recency: recently sick, not recently sick) mixed-model experimental design was used in Experiment 3, with illness recency being the between-subjects factor and category label being the within-subjects factor.

#### Materials and Procedure

Following previous studies (Miller and Maner, 2011; Lund and Miller, 2014; Prokosch et al., 2019), participants were categorized into two groups, a recently sick group (those who reported that they had been sick within the previous week) and a not recently sick group (those who reported that the last time they had been sick was more than 1 week ago). This categorization reflects the typical window of the biological system’s heightened susceptibility to new diseases after infection (Jakab, 1985; Miller and Maner, 2011; Lund and Miller, 2014; Murray et al., 2019). Participants in all groups were asked to take a bogus personality test at first and then to finish a face appraisal task. The bogus personality test which was used to create minimal groups and the face appraisal task that was employed by this experiment were identical to those of Experiment 1. After completing the face appraisal task, all participants were asked to complete the Perceived Vulnerability to Disease scale (PVD) (Duncan et al., 2009) to assess conscious concerns about disease. Participants responded to each item on a 7-point scale (with endpoints labeled “strongly disagree” and “strongly agree”). Following previous studies (e.g., Wu and Chang, 2012; Wu et al., 2015; Díaz et al., 2016; Liuzza et al., 2016), we used PVD as a single scale (*α* = 0.64) in Experiment 3. Higher scores on these measures indicate greater perceived vulnerability to diseases.

### Results and Discussion

**Figure 3:**
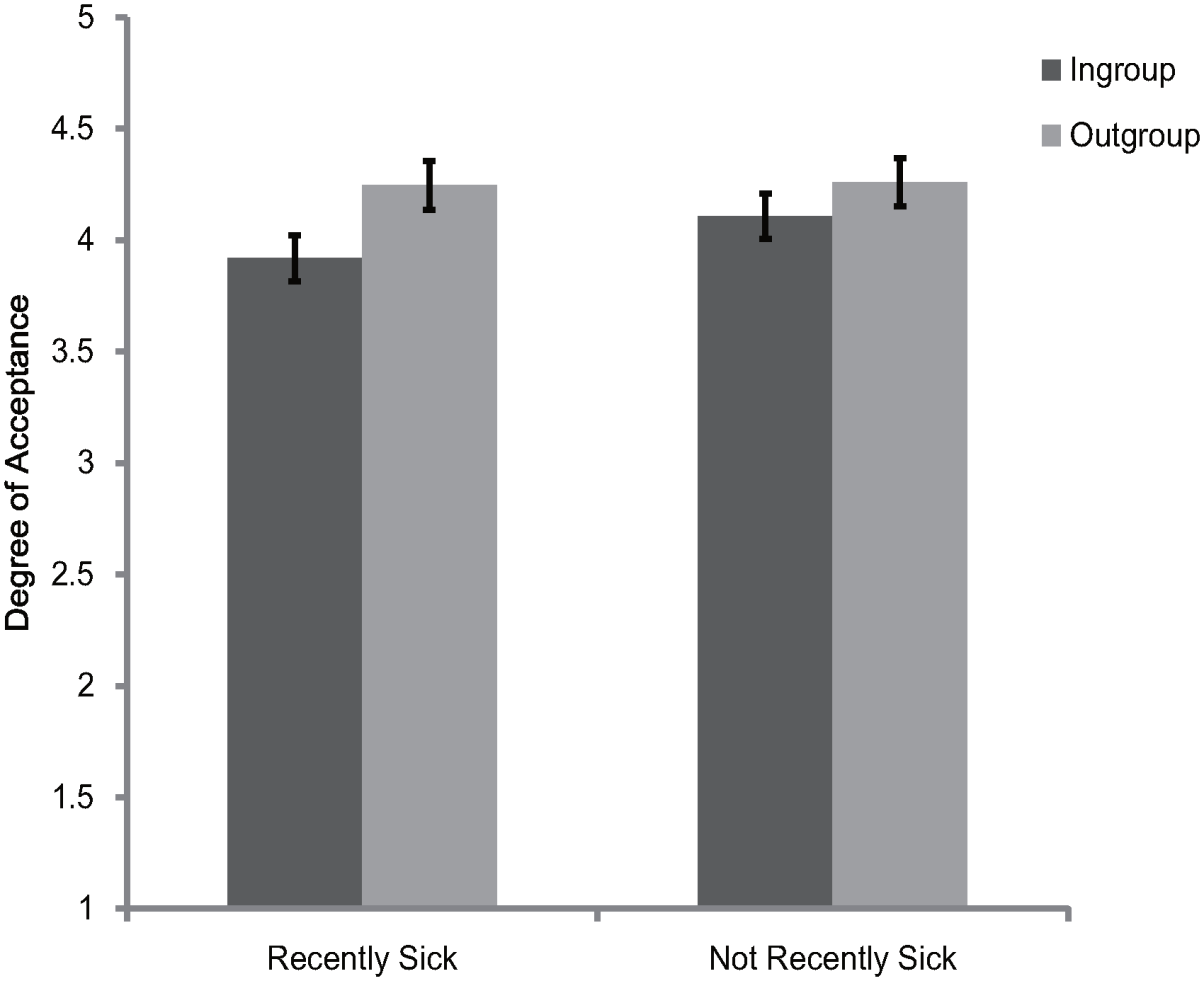
Degree of acceptance of faces labeled as ingroup members and outgroup members in Experiment 3. Error bars represent standard errors.

The 2 (category label) × 2 (illness recency) mixed model ANOVA on the rating scores of face appraisal task indicated that the main effect of category label [*F*(1, 120) = 44.82, *p* < 0.001, $\eta_{p}^{2}$ = 0.27] and the interaction between category label and illness recency [*F*(1, 120) = 6.09, *p* = 0.02, $\eta_{p}^{2}$ = 0.05] were significant. Consistent with Experiments 1 and 2, participants of the two illness recency groups consistently preferred the outgroup members over ingroup members [recently sick: *F*(1, 120) = 41.3, *p* < 0.001, $\eta_{p}^{2}$ = 0.26; not recently sick: *F*(1, 120) = 9.09, *p* = 0.003, $\eta_{p}^{2}$ = 0.07] (see Figure 3). The main effect of illness recency was not significant [*F*(1, 120) = 0.48, *p* = 0.49, $\eta_{p}^{2}$ = 0.004]. To better illustrate the interaction between category label and illness recency, the ingroup derogation score as described in Experiment 1 was created. Independent *t*-test showed, ingroup derogation attitudes were exaggerated for recently sick participants [recently sick: *M* = 0.33, SD = 0.35; not recently sick: *M* = 0.15, SD = 0.43; *t*(120) = 2.47, *p* = 0.02]. Further analysis revealed that the main effect of illness recency on ingroup derogation score remained significant even after controlling for PVD [*F*(1, 119) = 4.77, *p* = 0.03, $\eta_{p}^{2}$ = 0.04]. Thus, the results of Experiment 3 indicated that recent illness was accompanied by an exaggerated ingroup derogation tendency among Chinese participants. These results suggest that during a period of heightened susceptibility to new diseases after infection, the activation of ingroup derogation attitude would become stronger, and such effect was over and above the effects of overt concerns about disease vulnerability. Consistent with previous studies (Miller and Maner, 2011; Lund and Miller, 2014; Kandrik et al., 2017; Oaten et al., 2017; Gassen et al., 2018; Bradshaw et al., 2019; Murray et al., 2019), these results also suggest that the behavioral immune system will be more activated if the biological immune system is temporarily inhibited.

## General Discussion

In previous studies (e.g., Jost et al., 2002; Ashburn-Nardo et al., 2003; Zhao et al., 2012; Liu et al., 2015; March and Graham, 2015; Bettache et al., 2019), researchers mainly investigated the ingroup derogation phenomenon among actual social groups. With three behavioral experiments, the present study investigated the bias of ingroup derogation by using the minimal group paradigm. Although there were no real differences between the minimal groups, and no group members were labeled as deviants, the results of the three experiments in the current study still consistently showed that the Chinese participants derogated their ingroup members when they were asked to choose their partners purely based on the facial information and group membership. These results directly replicated the results of previous studies in which the Chinese participants were also found to be more inclined to cooperate with outgroup members under the minimal group paradigm (Wu et al., 2015, 2016). Similar results were also reported by researches using other tasks under minimal group paradigm (Zuo et al., 2018; Dang et al., 2019). For example, researchers found that East Asian participants allocated more resources to the outgroup members than to ingroup members when there were intragroup competitions within the minimal groups (Zuo et al., 2018). Taken together, the results of the current study showed that mere social categorization alone was sufficient to elicit ingroup derogation among Chinese participants, indicating that the ingroup derogation follows the smoke detector principle.

As an evolved response of behavioral immune system, except for being prone to make false-positive errors, the ingroup derogation also should follow the functional flexibility principle (Wu et al., 2015). Specifically, as a special adaptation to a particular situation in which ingroup members pose more threat of diseases than outgroup members, the ingroup derogation mechanism should modulate its responses accordingly when the individuals subjectively feel vulnerable to diseases (Condition 1), when there are cues of diseases in the immediate environment (Condition 2), when the relative risk of infection between ingroup and outgroup has been changed (Condition 3), or when the responses of biological immune system to new pathogens have been inhibited (Condition 4). The previous study had partially examined the functional flexibility of ingroup derogation under Conditions 1 and 2 (Wu et al., 2015). In the current study, we further investigated the functional flexibility of ingroup derogation under the last three conditions (Conditions 2, 3, and 4). In Experiment 1, we employed a more rigorous control to investigate whether Chinese participants would exaggerate their ingroup derogation attitudes after a disease prime (i.e., Condition 2) to rule out the alternative explanations that cannot be ruled out by the study of Wu et al. (2015). The results did show that the Chinese participants endorsed more ingroup derogation attitudes after a disease prime even when the overall affective valence and arousal were well matched between the disease prime and the non-disease-related threats control prime. In Experiment 2, we tested the Condition 3 by priming the Chinese participants with disease information about different social groups. The results indicated that, Chinese participants exaggerated their ingroup derogation attitudes after being primed with ingroup disease information (compared with a disease-related control prime which conveyed disease information about individuals with unknown group membership). The results also showed that, Chinese participants eliminated their ingroup derogation attitudes after receiving the disease prime which depicted the outgroup as infectious. In Experiment 3, we tested the Condition 4 by examining whether recent illness would promote the activation of ingroup derogation since fighting off one disease temporarily inhibits the physiological defenses against new diseases and consequently promotes the activation of behavioral immune system (Miller and Maner, 2011; Lund and Miller, 2014; Kandrik et al., 2017; Oaten et al., 2017; Murray et al., 2019). As predicted, the results showed that the ingroup derogation tendency was exacerbated when Chinese participants had been recently ill, and such effect was independent of the conscious concerns about disease. In summary, the results of the three experiments consistently indicate that the activation of ingroup derogation is related to the external disease cues (Experiments 1 and 2) and internal disease cues (Experiment 3). Collectively, these results suggest that ingroup derogation found among East Asian participants is an evolved response of behavioral immune system and it follows the functional flexibility principle.

While the previous study (Wu et al., 2015) suggests that ingroup derogation is a specialized mechanism which disregards explicit disease-relevant information mediated by outgroup members, a different pattern was observed in Experiment 2. Specifically, Experiment 2 showed that the participants eliminated their ingroup derogation attitudes after being primed with slides portraying medical cases of outgroup members. Consistent with the prediction made by previous study (Wu et al., 2015) in its discussion section (i.e., different pattern of results might be obtained if participants were separately facing the ingroup or outgroup members), the results of Experiment 2 was obtained by employing a between-subjects design in which the disease information of ingroup and outgroup members was separately presented (rather than be concurrently presented, as in Wu et al., 2015). Therefore, combined with the results of Wu et al. (2015), the results of Experiment 2 suggest that ingroup derogation is sensitive to the disease cues mediated by both ingroup and outgroup members, and they also suggest that the ingroup derogation mechanism will selectively respond to the disease cues mediated by ingroup members if the cognitive resources are getting depleted. Since the cognitive load was not manipulated either in the current study or in the study of Wu et al. (2015), this speculation still demands more investigation in the future. In addition, according to the evolutionary hypothesis of ingroup derogation, the ingroup derogation attitudes found among Chinese participants should be reversed to ingroup favoritism if the Chinese were primed with information depicting extremely strong outgroup disease threat. In Experiment 2, we only found that the ingroup derogation could be eliminated after receiving outgroup disease primes. Therefore, researchers still need to test this hypothesis by employing stronger disease threat primes. Cross-culture comparisons are also needed for a more thorough test of the evolutionary hypothesis of ingroup derogation. For example, researchers could try to prime the Western participants with ingroup disease primes to examine whether the ingroup favoritism attitudes can be reduced accordingly or be reversed to ingroup derogation attitudes.

In the previous study (Wu et al., 2015), researchers mainly focused on the effects of contextual cues. The current results provided the first empirical evidence for the hypothesis that temporary inhibition of the biological immune system facilitates the activation of ingroup derogation mechanism (as shown in Experiment 3). Recent studies have shown that the biological immune system and the behavioral immune system are connected on the cellular level, such as through the signals of proinflammatory cytokines (Il-6, Il-1β, and TNF-α), white cells, and stress and sex hormones (Kandrik et al., 2017; Gassen et al., 2018; Bradshaw et al., 2019; Murray et al., 2019). However, the current study was not designed to answer questions about the specific physiological mechanisms linking the biological immune system and the ingroup derogation mechanism. In addition, although we ruled out the effects of conscious disease concerns, we did not directly investigate the potential role of nonconscious goals. To clarify the biological and psychological processes mediating the link between the biological immune system and ingroup derogation, future research should address these limitations.

As a functionally coherent disease defense mechanism, the behavioral immune system can generate a series of consistent changes in down-stream perceptual, affective, cognitive, and behavioral processes (Schaller and Neuberg, 2012; Schaller et al., 2015; Murray and Schaller, 2016). Although previous studies have investigated the ingroup derogation phenomenon in East Asian cultures by using many different tasks, such as the face perception task (Jahoda et al., 1972; Zhao et al., 2012; Wu et al., 2016), emotion judgment task (Wu et al., 2016; Xie et al., 2019), memory task (Zhao et al., 2012), trait rating task (Ma-Kellams et al., 2011; Liu et al., 2015), attribution task (Hewstone and Ward, 1985), cooperation and allocation task (Wu et al., 2015, 2016; Zuo et al., 2018; Dang et al., 2019), etc., the current study had only examined the effects of infectious disease on ingroup derogation attitude in the domain of cooperation. If ingroup derogation is indeed an evolutionarily based disease defense mechanism, its activation should result in other functionally related changes, such as altered attention and avoidance response to threat-related targets (e.g., Miller and Maner, 2011). These questions demand further investigations in the future. In addition, in the current study, we mainly examined the effects of infectious disease on artificially constructed minimal groups. To thoroughly test the evolutionary hypothesis of ingroup derogation, we also have to examine these effects on natural social groups.

While the results of the current study suggest a potential link between disease threat and ingroup derogation in East-Asian cultures, the exact mechanisms that account for this link have not been identified by the present research. Although Experiment 2 showed that ingroup derogation could be modulated by specific environmental cues and thus it suggests that ingroup derogation is caused by the differential activation of functionally flexible neurocognitive mechanisms, other possible mechanisms still demand investigation (e.g., differential genetic selection and differential developmental trajectories). In addition, since the present studies only intended to offer an ultimate explanation for the ingroup derogation phenomenon, they are not able to offer any explanations in terms of proximate cause. It is entirely possible that the differential activation of behavioral immune system is proximately accomplished through the differential cultural transmission of learned behaviors (Chang et al., 2011; Scott-Phillips et al., 2011; Lewis et al., 2017). Currently, the only plausible proximate explanation for the ingroup derogation phenomenon found in East Asian cultures is the dialectic theory in which researchers proposed that individuals with East Asian culture background are inclined to appraise both bad and good for the same object (Ma-Kellams et al., 2011). However, this theory can only explain why the criteria of appraisal for East Asians are stricter, but it cannot explain why East Asian participants still derogated their ingroup members when they held the same dialectical belief toward both ingroup and outgroup members (Zhao et al., 2012; Wu et al., 2015, 2016). Can other cultural-specific factors (e.g., different value emphases, different social relationships; for review, see Chang et al., 2011) mediate or moderate the relationship between disease threat and ingroup derogation? Researchers should look into this question in the future. In addition, it should also be noted that while we mainly investigated the ingroup derogation phenomenon among mainland Chinese participants, the current findings are not directly applicable to the ingroup derogation found in minority groups (e.g., Allport, 1958; Jost et al., 2002; Livingston, 2002; Rudman et al., 2002; Ashburn-Nardo et al., 2003; Umphress et al., 2008; March and Graham, 2015; Axt et al., 2018) or to the ingroup derogation found against deviant ingroup members (Marques et al., 1988; Kunstman et al., 2016; Bettache et al., 2019). Although the theory of behavioral immune system may offer the ultimate explanation for the black sheep effect, explaining the ingroup derogation found in socially disadvantaged groups would be another story (e.g., Wu et al., 2015, 2016).

Previous studies on ingroup favoritism mainly support the theory that ingroup favoritism is an adaptive response from the behavioral immune system (Fincher and Thornhill, 2008a,b, 2012a,b; Van Vugt and Park, 2009; Schaller and Murray, 2010; Neuberg et al., 2011; Schaller and Neuberg, 2012; Thornhill and Fincher, 2014; Schaller et al., 2015; Murray and Schaller, 2016; Neuberg and Schaller, 2016; Ji et al., 2019; Zakrzewska et al., 2019). However, some recent studies have also found that the negativities toward outgroups may not be an adaptive outcome but a byproduct of the behavioral immune system. That is, the behavioral immune system is sensitive to any type of deviation and the outgroups happen to look dissimilar (Petersen, 2017; van Leeuwen and Petersen, 2018). By demonstrating that the behavioral immune system is sensitive to source of disease threat in Experiment 2, the current study supports the adaptation account and suggests that the behavioral immune system contains perceptual mechanisms for which some features that correlate with ingroup and outgroup memberships are part of proper domain. However, we should also be noted that the adaptation account and the byproduct account are not necessarily exclusive to each other and actually both of these two causes may contribute to the intergroup bias we found in human societies (Ji et al., 2019). In fact, by the results of Experiment 2 alone, we are not able to completely rule out the possibility that the byproduct cause also contributes to the ingroup derogation we found in the current study. That is, due to the low inter-regional mobility within East Asian countries and the high pathogen loads faced by East Asians (Chang et al., 2011), the behavioral immune system of East Asian participants is calibrated to detect the dissimilarities between ingroup members which makes them become less tolerant toward their ingroup members. This possibility should be tested by future works.

The present work adds more evidence to the disease prevalence account of cultural differences (Fincher et al., 2008). Previous studies mainly focused on the social behaviors on this aspect. For example, researchers have found that nations with greater pathogen loads are more religious, more collectivistic, more likely to conform, more conservative, etc. (Fincher et al., 2008; Schaller and Neuberg, 2012; Thornhill and Fincher, 2014; Schaller et al., 2015; Murray and Schaller, 2016). By testing the effects of disease threat on the ingroup derogation attitude among Chinese participants, the current study suggests that East Asians are responding to a special ecological condition in which in which the greater threat of diseases is incurred by ingroup members and thus they may have a unique pattern in their activation of behavioral immune system. Given the importance of behavioral immune system in shaping our basic cognitions (e.g., Miller and Maner, 2011, 2012; Makhanova et al., 2015; Liuzza et al., 2016; Murray and Schaller, 2016; Nussinson et al., 2018; Bonin et al., 2019; Prokosch et al., 2019; Wang and Ackerman, 2019), we may expect to find other functionally related differences between Easterners and Westerners in the processes of perception (Nussinson et al., 2018), memory (Bonin et al., 2019), emotion (Xie et al., 2019), decision (Prokosch et al., 2019), etc. These are important directions for future research.

## Conclusion

Ingroup derogation is a counterintuitive phenomenon that apparently contradicts both expert and lay beliefs. The current findings suggest that the activation of ingroup derogation mechanism is related to external environmental disease cues and internal physiological disease cues. Such a mechanism is also sensitive to the specific perceived vulnerabilities to ingroup disease threat and outgroup disease threat. Thus, the current research supports the evolutionary hypothesis of ingroup derogation and suggests that the ingroup derogation found in East Asian cultures may be explained by a functionally flexible disease-avoidance mechanism.

## Data Availability

The datasets generated/analyzed for this study can be found in the figshare: https://figshare.com/s/f811b11f9e17121346cc. Password: 20190628hunnu.

## Ethics Statement

The studies involving human participants were reviewed and approved by The IRB of the Institute of Psychology, Hunan Normal University. The patients/participants provided their written informed consent to participate in this study.

## Author Contributions

QW and PZ conceived and designed the experiments. QW and SY performed the experiments and analyzed the data. QW drafted the paper. All the authors participated in the revising of the paper, approved the version’s publishment, and agreed to be accountable for all aspects of the work.

### Conflict of Interest Statement

The authors declare that the research was conducted in the absence of any commercial or financial relationships that could be construed as a potential conflict of interest.

## Footnotes

^1^Some of the studies on ingroup favoritism mainly focused on the aspect of outgroup negativity, whereas some of the studies mainly focused on the aspect of ingroup preference. To simplify the discussion, in the present research, we call all these related phenomena as ingroup favoritism.

^2^In the minimal group paradigm, the targets who belong to the same assigned category of participants are called ingroup members, whereas the targets who do not belong to that category are called outgroup members (Tajfel et al., 1971; Brewer, 1979; Bernstein et al., 2007; Paladino and Castelli, 2008; Montalan et al., 2012; Makhanova et al., 2015).

^3^We employed the mean effect size in social psychology (Richard et al., 2003) as our estimation of effect size.
